# A large-effect locus underlies migration timing in North American Atlantic salmon (*Salmo salar*)

**DOI:** 10.1038/s41598-026-42281-w

**Published:** 2026-03-02

**Authors:** Samantha V. Beck, Tony Kess, Cameron M. Nugent, Brian Dempson, Gerald Chaput, Steve Duffy, Nicole Smith, Paul Bentzen, Victoria L. Pritchard, Ian R. Bradbury

**Affiliations:** 1 Institute for Biodiversity and Freshwater Conservation, University of the Highlands and Islands, Inverness, Scotland; 2 Fisheries and Oceans Canada, Northwest Atlantic Fisheries Centre, St. John’s, NL Canada; 3 Biology Department, Dalhousie University, Halifax, Canada; 4 Fisheries and Oceans Canada, Moncton, NB Canada

**Keywords:** Evolutionary genetics, Animal migration, Biodiversity

## Abstract

The timing of migration often aligns with predictable seasonal or environmental cues, allowing populations to maximise fitness by moving between habitats at optimal times. However, rapid environmental change is disrupting this predictability, leading to mismatches between expected and observed conditions with potential demographic consequences. Atlantic salmon are long-distance migrators that travel between freshwater and oceanic habitats and are experiencing widespread declines across their range. Our understanding of the genetic basis of run timing in Atlantic salmon has been limited to European populations, or at a coarse population level. We combine whole-genome sequencing of 498 individuals from seven North American populations with individual migration timing data to explore the genomic basis of adult return timing. We identify a large-effect region on chromosome 17 associated with migration timing (unimodal or bi-model), with *ppfia2* explaining a substantial proportion of the variation, as well as an underlying polygenic basis to this complex life-history trait. These findings demonstrate a clear genomic basis for migration timing in Atlantic salmon, with the associated *ppfia2* gene also playing a role in other long-distance migratory vertebrates. This suggests a potentially conserved evolutionary mechanism underlying migration timing across species and highlights the importance of genetic insights for understanding population resilience and declines in Atlantic salmon.

**Supplementary Information:**

The online version contains supplementary material available at 10.1038/s41598-026-42281-w.

## Introduction

Seasonal migration is a widespread phenomenon observed in numerous taxa, where populations fine-tune their movements through adaptive evolution to align with predictable seasonal cues, maximising fitness and reproductive success in temporally productive habitats. As such, understanding the genetic basis of complex life-history traits such as migration timing can have important implications for the conservation of wild populations^1^. Yet the genetic architecture of migration timing remains poorly understood in many species, despite mounting evidence demonstrating how migration timing is changing in response to rapid environmental change^2–11^. Ultimately, populations’ evolutionary potential and persistence in the face of climate change will partly depend on their ability to adapt and track changing environmental conditions^12,13^.

This capacity for evolutionary response to climate change will be determined by the strength of selection, the heritability, and genetic architecture of the traits in question^14^. Most complex phenotypic traits have genetic associations spread across the genome^15^, with adaptive change driven by shifts in allele frequencies across numerous loci (e.g. height^16^. However, not all complex traits are polygenic, with recent genomic studies identifying single regions of large effect in multiple species. For example, songbird migration timing has been localised to single genomic region in two species of warblers (*Vermivora chrysoptera* and *V. cyanoptera*;^17^. This region contains *VPS13A* (Vacuolar Protein Sorting 13 Homolog A), a gene involved in mitochondrial function^18^ and lipid transport^19^, which, although speculative, may help mitigate oxidative stress during prolonged migratory flights^17,20^. In salmonids, premature migration timing in Chinook salmon (*Oncorhynchus tshawytscha*) and anadromous rainbow trout (Steelhead; *O. mykiss*) has been repeatedly associated with the *GREB1L* (growth regulation by estrogen in breast cancer 1-like)*/ROCK1* (Rho Associated Coiled-Coil Containing Protein Kinase 1) region^21–23^. Anthropogenic impacts are reducing the frequency of the *GREB1L* allele associated with earlier migration in Chinook salmon, inducing rapid evolution primarily through genetic change as opposed to phenotypic plasticity^23^. The Chinook salmon spring-run allele arose in a single mutational event, so if lost from the population, it is unlikely to evolve again within conservation timescales^22^. These examples highlight the importance of maintaining genetic diversity within populations to preserve adaptive potential and enable evolutionary responses to changing environmental conditions.

Migration timing in ectotherms, such as fish, may be particularly sensitive to environmental changes due to their inability to regulate internal temperatures. Fish can exhibit behavioural thermoregulation to cope with temperature fluctuations, displaying daily rhythms of thermal preference^24^. Beyond short-term behavioural responses, considerable diversity exists in migration timing—or run timing—across fish species and populations^6,25–29^. This diversity in migration phenotype of fishes can be attributed in part to multiple whole-genome duplication (WGD) events, resulting in an abundance of genes unconstrained to selective pressures^30^. One example of this is in salmonids, where four rounds of WGD (Ss4R) preceded the evolutionary development of novel complex migratory behaviours^31^. Whilst many of these duplicated genes were lost and silenced, gene duplication provided a source of new genetic material, enabling the evolution of novel functions and contributing to a rich complexity of genes^32^, including those associated with circadian rhythms (i.e., clock genes;^33^. More specifically, clock genes have been found to mediate seasonal adaptation and variation in reproductive timing in some species of Pacific salmon^34^ with variation at a single locus (*OtsClock1b*; a salmon-specific version of the *Clock* gene, a fundamental component of the molecular circadian clock^35^ associated with timing of adult returns in Atlantic salmon (*Salmo salar*^36^.

Atlantic salmon is a predominantly anadromous salmonid distributed throughout the North Atlantic, whose populations often migrate long distances to northern feeding grounds at sea and back again to natal rivers to spawn (e.g.^37^. These long-distance migrations are vulnerable to climate variability with mismatches between local and destination conditions a potential factor contributing to their ongoing declines^38,39^. Although some Atlantic salmon populations appear to be shifting their migration timing to correspond with changes in the climate^3,40^, our understanding of its genetic basis is limited to SNP array data and only a few European populations^41–43^ or at a population-level^44^. These studies identify a genomic region near *six6* (sine oculus-related homeobox 6) that influences the timing of adult salmon returns, a gene that is also associated with age-at-maturity (a correlated trait influencing when adults start their return to native rivers to spawn), alongside the large-effect *vgll3* (vestigal-like protein 3) locus^43,45–47^. In addition to *six6*, Beck et al.^44^ also finds *vgll3* and other genes linked with age-at-maturity associated with early migration timing, suggesting some degree of parallelism in life-history traits. A further example is how the *six6-lrrc9* (leucine rich repeat containing 9) region has also been found associated with life-history diversity in multiple species of Pacific salmon^48,49^, including migration timing^50^. The underlying genetic basis of phenological traits closely intertwined with the environment (e.g. migration timing) can ultimately influence reproductive success and determine the overall capacity of populations to adapt to novel climates.

Although these studies have provided valuable initial insights into the genetic basis of migration timing in European Atlantic salmon, significant gaps remain, particularly in regard to North American Atlantic salmon. To address this knowledge gap, this study uses low-coverage whole genome sequencing (lc-WGS) and individual-level run timing data, offering a more detailed transatlantic understanding of the genetic underpinnings of migration timing. Our study builds upon prior work exploring the impacts of climate change on migration timing^3,40^, and its genetic foundations^41–44^, but goes further by providing the most comprehensive examination of the genomic basis of this trait in Atlantic salmon to date. By demonstrating a clear genomic basis for migration timing and illustrating the conservation of this genomic architecture across a diverse suite of taxa, our study contributes new insights into the genetic mechanisms underlying migration timing and the potential for adaptive responses to environmental shifts.

## Methods

### Individual run timing descriptors and genotyping

**Fig. 1:**
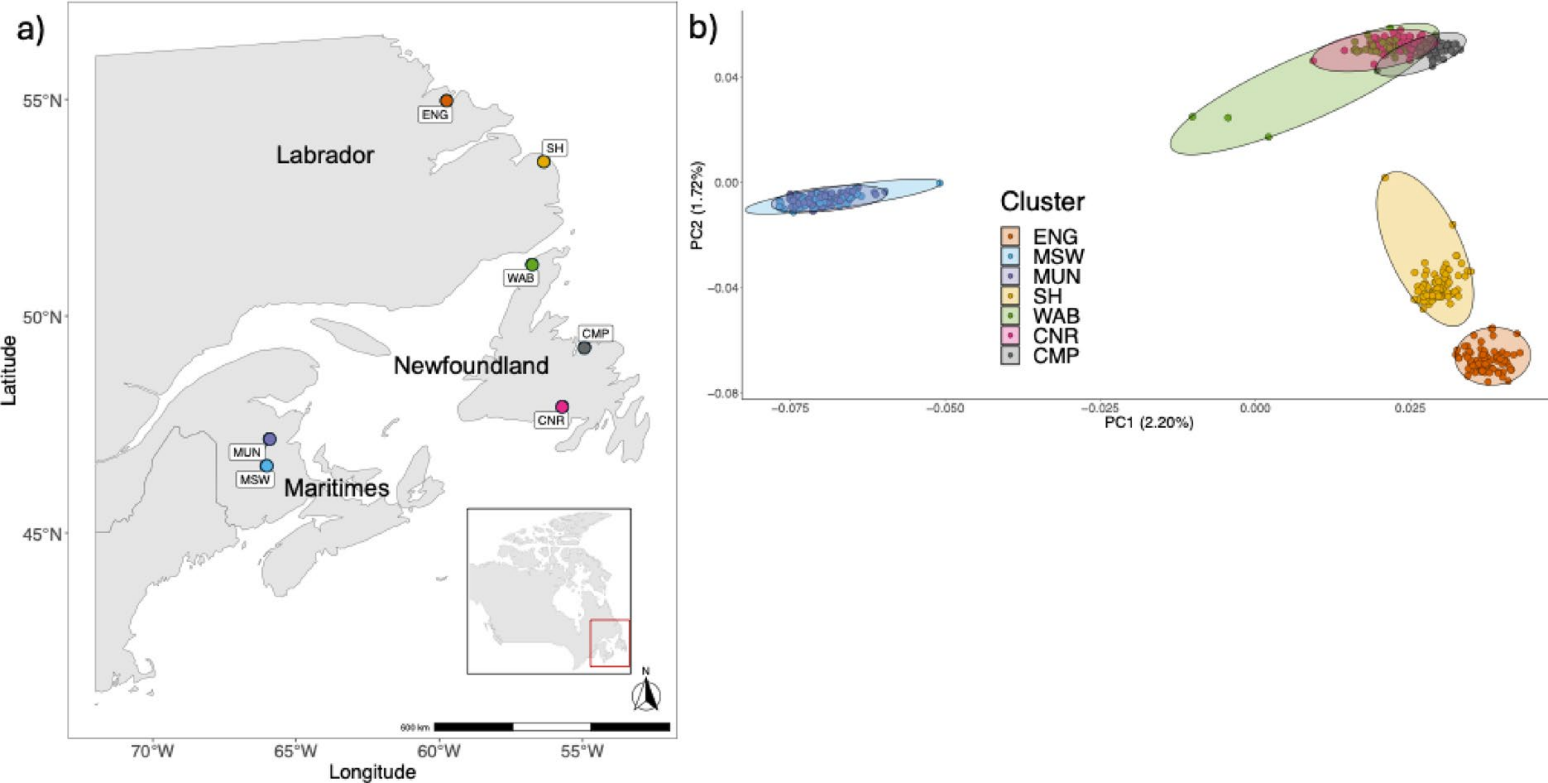
(**a**) Locations of 7 North American Atlantic salmon populations and (**b**) principal component analysis (PCA) of genetic variation showing population structure for the 7 populations (Maritimes = MSW, Southwest Miramichi; MUN, Miramichi Upper Northwest; Newfoundland = CMP, Campbellton; CNR, Conne River; WAB, Western Arm Brook; and Labrador = SH, Sand Hill; ENG, English River) of North American Atlantic salmon, using the first two PC axes.

**Fig. 2:**
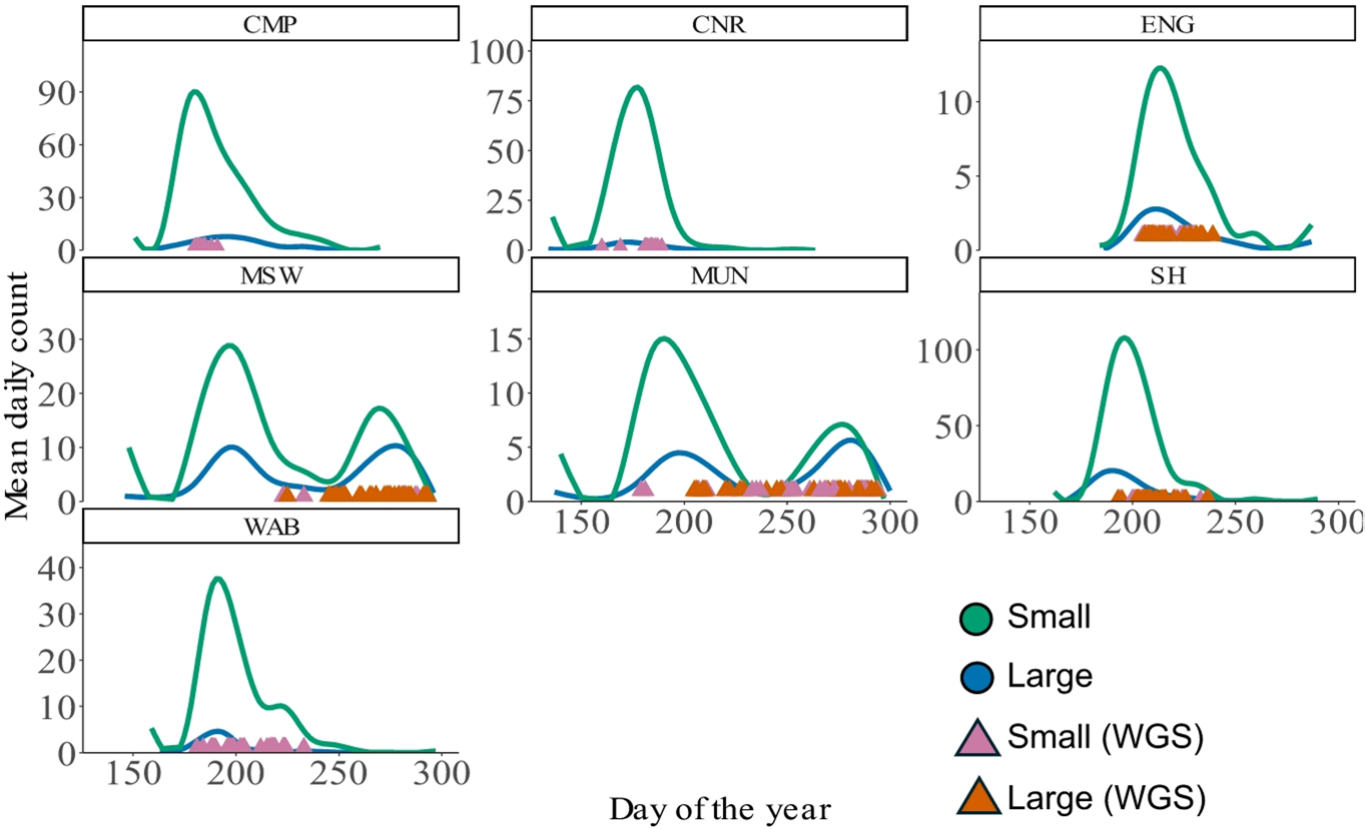
Mean daily count of North American adult Atlantic salmon returns across a 29-year period (1993–2021), coloured according to size (blue = large (> 63 cm), green = small (< 63 cm)). Samples used for low-coverage whole genome sequencing (WGS) are represented by triangles and coloured pink (small < 63 cm) and orange (large > 63 cm) to show where in the run timing distribution they were sampled from. Maritimes = MSW, Southwest Miramichi; MUN, Miramichi Upper Northwest; Newfoundland = CMP, Campbellton; CNR, Conne River; WAB, Western Arm Brook; and Labrador = SH, Sand Hill; ENG, English River.

We explored the relationship between run timing and genotype at the individual level using fish sampled in seven out of the 11 populations examined by Beck et al.^44^. These comprise English River (ENG) and Sand Hill River (SH) in Labrador, Miramichi Southwest (MSW) and Miramichi Upper Northwest (MUN) in the Maritimes, and Conne River (CNR), Western Arm Brook (WAB) and Campbellton (CMP) in Newfoundland (Fig. 1a). All fish were caught <6 km from the river mouth (Table 1), with samples collected mostly around the peak migration window (Fig. 2). A total of 498 individuals were used in this study comprising of 222 males and 276 females (Table 1). For each individual, we used day of the year (DOY) of capture as our run timing measure (Fig. 2).

**Table 1:** Sample table describing each trapping location, approximate distance of trap from estuary (km), and number of individuals (n) collected by sex and age at maturity (one sea-winter, 1SW; multi-sea-winter, MSW), along with the total number of samples genotyped using low coverage whole genome sequencing.

Population	Code	Region	Distance from estuary (km)	Latitude	Longitude	Female (*n*)	Male (*n*)	1SW (*n*)	MSW (*n*)	Total (*n*)
Campbellton River	CMP	Newfoundland	0.5	49.26852	-54.92938	29	36	65	0	65
Conne River	CNR	Newfoundland	2	47.92333	-55.68333	35	6	41	0	41
English River	ENG	Labrador	0.5	54.96667	-59.75	46	32	47	31	78
Miramichi Southwest	MSW	Maritimes	0	46.88167	-65.66	29	28	21	36	57
Miramichi Upper Northwest	MUN	Maritimes	0	46.93667	-65.77833	45	41	52	34	86
Sandhill	SH	Labrador	6	53.5592	-56.36733	46	43	46	43	89
Western Arm Brook	WAB	Labrador	0.5	51.19	-56.76833	46	36	82	0	82

Low-coverage whole genome sequencing (lc-WGS) was conducted for all individuals, as detailed in^51^. In brief, genomic DNA was amplified using Kapa Hi-Fidelity Library Amplification Kits (Roche) with Nextera Unique Dual Indexes Set A (Illumina), quantified using Qubit (ThermoFisher), normalised and then sequenced using Illumina NovaSeq6000 S4 at the Genome Quebec Centre d’Expertise et de Services. Quality of sequenced libraries was checked using FastQC^52^, reads were trimmed to remove leading 15 bases, adapter content, bases falling below of q score of 10, and any read with less than 40 remaining base pairs^53^ and aligned to the 29 chromosomal contigs from the ICSASG_V3.1 *Salmo salar* reference assembly (GCA_905237065.2)^32^ using *bwa mem* 0.7.17^54^. Duplicate reads were removed using PicardTools 2.20.6 (http://broadinstitute.github.io/picard/) MarkDuplicates function, and sorted and realigned around potential insertions and deletions using RealignerTargetCreator and IndelRealigner functions in GATK^55^.

Methods for genotype calling are described in detail in Kess et al.^51^, but to summarise: genotype likelihoods, as well as directly called genotypes, were inferred for each individual using Analysis of Next Generation Sequencing Data (ANGSD 0.935^56^. SNPs were filtered to retain only those with genotype likelihoods > 80% across all sequenced individuals and a minimum of 500 reads per locus. The mean depth of coverage for each SNP was assessed across all individuals using vcftools 0.1.16^57^ to ensure the reliability of variants. Missing genotypes were then imputed using Beagle 4.0^58^, resulting in 9,789,862 SNPs with a mean coverage of 4.11x across all individuals for downstream analysis.

### Population structure

To avoid any disproportionate influence of regions of high LD such as chromosomal rearrangements in our assessment of population structure, we first applied a linkage disequilibrium (LD) filter with the PLINK v1.9^59^ parameter --indep-pairwise 50 5 0.5 (window size of 50 SNPs, shifting the window 5 SNPs at a time, with a correlation threshold of r^2^ = 0.05 between SNP pairs) to get independent SNPs (*n* = 1,821,355). Individuals with a heterozygosity rate deviating more than 3 standard deviations (SD) from the mean using the --het flag(*n* = 1) were removed, as such deviations may indicate underlying quality issues such as contamination or abnormal relatedness. For each population, likely first-degree relatives (kinship coefficient > 0.177) were removed (*n* = 18) after application of PLINK’s (v2.0) KING-robust kinship estimator. PLINK’s *--pca* parameter was also used to explore population structure using principal components analysis (PCA), with the most suitable number of PCs inferred from examination of scree plots using the ‘broken stick approach’^60^.

### Genotype associations with individual-level run timing

Run timing associations with individual WGS data were conducted in *PLINK* 1.9 following a protocol by Marees et al.^61^, without correcting for population structure, as well as inclusion of the first three PC axes as covariates to correct for population structure. To ensure that finer-scale population substructure was not influencing the results, we also repeated the analysis including the first 20 PC axes. We used the --linear option in PLINK to perform a linear regression analysis with each SNP as a predictor, including sex and age-at-maturity as covariates (--covar), for both the population structure corrected and uncorrected GWAS. A Benjamin-Hochberg^62^ false-discovery rate (FDR) was applied (--adjust) to reduce the number of false-positives from our associations. Chromosomal position and gene overlaps of candidate SNPs were identified using the --intersect function from Bedtools^63^ and the annotated Atlantic salmon genome (v3.1^32^. Finally, we identified potential enrichment of associated genes in genetic pathways using the Ssal_v3.1 Atlantic salmon genome as the genomic background in ShinyGO 0.80^64^. Gene IDs were mapped to Ensembl and STRING-db protein identifiers, and enrichment was tested against pathway information compiled from public databases, including KEGG (https://www.genome.jp/kegg/^65^ and Gene Ontology (GO) Biological Processes (https://geneontology.org^66^. Fold enrichment values indicate the ratio of observed to expected gene counts within a given pathway, with higher values reflecting stronger overrepresentation among the associated genes.

## Results

### Population structure

Analysis of population structure showed that most variation was explained by the first three PCs. PC1 (2.20% of the variation) separated the Maritimes’ populations, and PC2 (1.72%) distinguished populations from Newfoundland and Labrador (Fig. 1b). The first seven PCs together captured the main axes of differentiation within the pruned SNP dataset (Fig. S1a). PC3 (0.94%) further separated populations within Newfoundland (WAB from CNR and CMP; Fig. S1b). PCs 4–7 each explained less than 1% of the variance, and all remaining PCs explained < 0.3%.

### Genotype associations with individual-level run timing

**Fig. 3:**
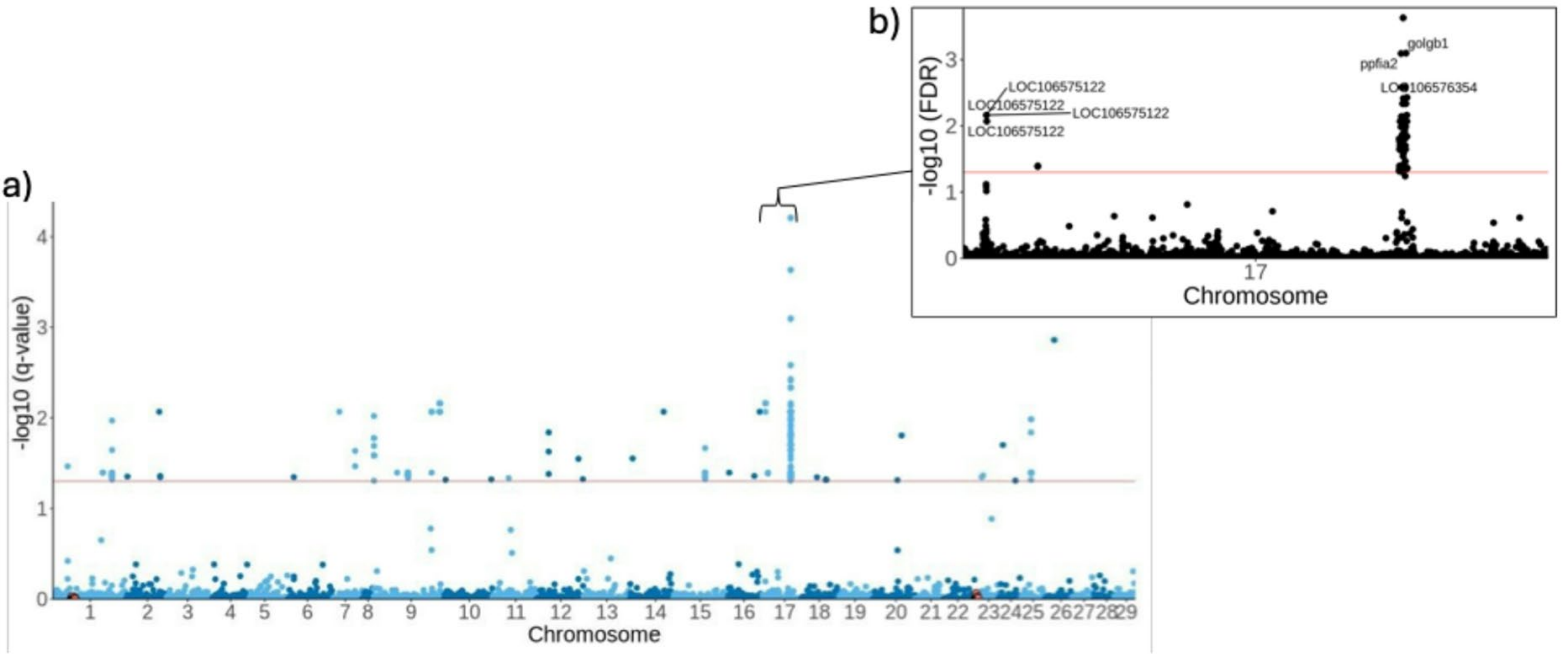
Manhattan plot (**a**) showing associations with individual adult return in 7 populations of North American Atlantic salmon, with SNPs above the red line representing outliers, whilst inset plot (**b**) highlights associations on chromosome 17.

When examining associations with day of individual sampling, correcting for sex and age-at-maturity but not population structure, we found 2,980,691 unique SNPs overlapping 42,198 unique genes. After correcting for population structure using 7 PC axes, a total of 193 unique SNPs were associated adult return timing (including those in non-coding regions; Table S1), 105 of which were overlapping genes (*n* = 32; Table S2). Whilst primarily concentrated on chromosome 17, other outliers exist that were scattered across the genome, with peaks evidence on chromosomes 1, 8, 9 and 15 (Fig. 3a). The highest outlier on chromosome 9 was *cadm1b* (Cell Adhesion Molecule 1), but we find no evidence of associations with, or near, *six6.* An entire region on Ssa17 spanning 109,044 bp (63,670,009–64,737,682 bp) seems to strongly associated with adult return timing, consisting of *ppfia2* (PPFI Scaffold Protein A2), *golgb1* (Golgin B1), *lhfpl3* (LHFPL Tetraspan Subfamily Member 3), *llph* (LLP Homolog, Long-Term Synaptic Facilitation Factor) and *chrm2a* (Cholinergic Receptor Muscarinic 2). Whilst the highest association found across all loci was in a non-coding (63,670,009 bp) region (Table S1), the strongest gene-overlapping signal, *ppfia2*, was found just downstream (63,638,643) (Table S2), which alone explained 31.4% of the variation in individual day of adult returns. The strong association with *ppfia2* remained after accounting for fine-scale population structure using 20 PCs (Table S3).

Genes associated with individual day of return after correcting for population structure were significantly enriched in 7 genetic pathways due to a single gene, *chrm2a* (Ssa17). All pathways were associated with regulating cardiac function and circulatory processes (Table S4).

## Discussion

Timing and duration of species movements can have important consequences on fitness and survival^2,67–69^, imposing strong selective pressures on a trait evolved alongside historically predictable climates. However, climate change increasingly disrupts this alignment, creating mismatches that can drive evolutionary shifts in migration-related genes^70^. Through whole genome sequencing of North American Atlantic salmon, we have identified a genomic region on chromosome 17 that is strongly linked to the timing of adult salmon return migration. This region overlaps *ppfia2*, a gene previously associated with migration timing in a long-distance migratory bird (purple martin, *Progne subis subis*)^71^. The highest observed association was found within a non-coding region just upstream of *ppfia2*, suggesting that gene regulation may play an important role in modulating migration timing in relation to shifting environmental cues, such as temperature or photoperiod. Consistent with this, GO enrichment identified cardiac function–related pathways, which may reflect physiological responses to temperature or oxygen availability. This study not only deepens our understanding of the genomic basis and potential regulation of migration timing in Atlantic salmon but also suggests that *ppfia2* may play a conserved role in migration timing of vertebrates more broadly.

### Conserved gene underlying migration timing across vertebrates.

Previous studies in European Atlantic salmon found evidence for the association of *six6* with run timing^41–43^. Here, in North American Atlantic salmon, we find no evidence of associations with *six6*, despite its involvement in ocular development^72–74^, circadian rhythms^75,76^ and reproduction^77,78^. Instead, we find strong associations with *ppfia2* (Ssa17), likely driven by the diversity of run timing present in the Maritimes (Fig. 2). Whilst this study lacks the statistical power to test for associations with run-timing modality, Beck et al.^44^ was able to specifically test for such associations using an Atlantic salmon 220k SNP dataset spanning the same and additional populations, identifying *ppfia2* as associated with modality and *six6* with run timing. Bi-modal migration timing patterns were similarly observed in the study on purple martins, which also identified strong associations with *ppfia2*^71^. Such a conserved mechanism suggests a critical role of *ppfia2* in regulating life-history traits, where it likely provides a stable mechanism for adaptation to environmental conditions and trade-offs that have remained advantageous over evolutionary time. To add further support to this hypothesis, a copy of *ppfia2* on chromosome 7 in Atlantic salmon has been shown to have reduced heterozygosity in anadromous compared to landlocked Atlantic salmon populations, suggesting relaxed selection in landlocked populations that no longer migrate to sea^79^. This study is one of very few that documents a shared mechanism linking life-history traits across taxa^80^, offering important insights into the evolutionary processes that govern these traits.

### Large-effect locus, polygenicity and regulatory effects.

Migration timing is a complex life-history trait influenced by multiple environmental, physiological and behavioural factors^81^. The timing of migratory events has not evolved in isolation, with multiple correlated traits implying that migration is a ‘syndrome’ that has evolved in synchrony with predictable environmental cues^82^. As such, the direction of energy and nutrients towards one trait comes at the trade-off of another, leading to evolutionary constraints of this important life-history trait, as well as extensive individual variation in both phenotype and genotype^82^. Given the complexity of migration timing and its interaction with various life-history traits, it is likely that multiple genes beyond the large-effect locus of *ppfia2*, also contribute to the regulation of this trait (e.g.^47^, each exerting smaller, yet significant, effects in collectively shaping the timing of migration. For example, *cadm1b*, the highest-associated locus on Ssa9 in our analysis, and *cadm1* on Ssa13, a paralogue, has been previously linked to the divergence between anadromous and landlocked Atlantic salmon^79^. These findings underscore the importance of both large-effect loci, like *ppfia2*, and the collective contributions of smaller-effect genes in shaping the polygenic basis of migration timing. Further investigation into the role of *ppfia2* and other associated loci is essential, as understanding this complex network of genetic factors could be key to predicting how populations may respond to climate change, particularly through the generation and maintenance of diverse migration timing strategies that buffer populations against environmental variability^27,83^.

Here, we find that the highest SNP associated with migration timing was located in a non-coding region just upstream of *ppfia2*, whereby variants within this region could influence promoter activity, transcription factor binding, or the formation of enhancers, potentially controlling the timing and level of *ppfia2* expression^84,85^. By acting as a regulatory element, this region could provide a flexible mechanism crucial for modulating gene expression in response to rapid environmental change or other stimuli, playing a critical role in fine-tuning migration timing. An important component underlying plastic responses to environmental change is regulatory variation, which enhances the phenotypic flexibility of a single genotype by fine-tuning gene expression through epigenetic mechanisms and non-coding regulatory elements such as enhancers, silencers and non-coding RNA^86–88^. Not all individuals or genes have the same sensitivity to environmental signals^89^, which can result in alternative phenotypic outcomes under similar conditions^90,91^.

### Challenges and future directions.

Increasing our understanding of the genomic basis of migration timing is particularly relevant given ongoing changes in environmental conditions that are expected to affect phenological traits^92,93^. Now that we have identified key genetic associations with this climate-linked trait, future studies can not only explore this pattern within other species with known bi-modal migrations but also build on these findings to assess how different genetic architectures may influence population responses to shifting climate regimes. Such insights would improve our understanding of the adaptive capacity in phenological traits and, when combined with appropriate modelling frameworks, contribute to predictions of species responses to environmental change and their implications for conservation.

Whilst this study focused on the genomic basis of migration timing, further investigation is needed to characterise the regulatory mechanisms that may influence the phenotype. In particular, investigating how *ppfia2* expression is regulated across populations with differing run timing phenotypes could help clarify whether regulatory variation at the *ppfia2* locus contributes to phenological diversity through differences in gene expression. Such analyses may be especially informative in regions such as the Maritimes, where multiple run timing peaks occur. This study lacked the power to explore patterns of bi-modality at the individual level, as matched run timing and genetic data were only available for seven populations. However, population-level analyses were able to associate *ppfia2* with bi-modal run timing^44^, demonstrating the value of complementary and historical population-scale datasets for uncovering the genetic basis of complex migration patterns.

## Conclusion

This study provides new insights into the genetic basis of migration timing in North American Atlantic salmon, identifying *ppfia2* as a candidate gene strongly associated with run timing. The identification of a significant non-coding region upstream of *ppfia2* suggests that gene regulation may play a crucial role in shaping phenological differences, potentially through effects on gene expression rather than protein-coding change. The association of *ppfia2* with migration timing in both Atlantic salmon and long-distance migratory birds^71^ points to a conserved genomic region underlying this complex life history trait. Although migration timing is influenced by many genetic and environmental factors, our results highlight the potential importance of large-effect loci and regulatory elements in contributing to phenological diversity. Future research integrating genomic, regulatory, and expression-based approaches will be essential to clarify the mechanisms linking genetic variation at the *ppfia2* locus to variation in migration timing across populations, particularly where shifts in phenology may have important ecological consequences.

## Supplementary Information

Below is the link to the electronic supplementary material.

Supplementary Material 1

## Data Availability

New whole genome sequencing data used in these analyses are available in the ENA under accession PRJEB111809. Whole genome data from Kess et al. (2024) used here are available in the ENA with accession number PRJNA1083490. All other data are available at 10.5281/zenodo.20793856.

## References

[1] Waples, R. S. et al. Implications of large-effect loci for conservation: A review and case study with Pacific salmon. *J. Hered.* **113** (2), 121–144. 10.1093/jhered/esab069 (2022). 35575083 10.1093/jhered/esab069

[2] Acácio, M. et al. Timing is critical: consequences of asynchronous migration for the performance and destination of a long-distance migrant. *Mov. Ecol.* **10**, 28 (2022). 35725653 10.1186/s40462-022-00328-3 PMC9901525

[3] Dempson, B. et al. Influence of climate and abundance on migration timing of adult Atlantic salmon (*Salmo salar*) among rivers in Newfoundland and Labrador. *Ecol. Freshw. Fish*. **26** (2), 247–259. 10.1111/eff.12271 (2017).

[4] Gienapp, P., Leimu, R. & Merilä, J. Responses to climate change in avian migration timemicroevolution versus phenotypic plasticity. *Clim. Res.* **35**, 25–35 (2007).

[5] Juanes, F., Gephard, S. & Beland, K. F. Long-term changes in migration timing of adult Atlantic salmon (*Salmo salar*) at the southern edge of the species distribution. *Can. J. Fish. Aquat. Sci.* **61** (12), 2392–2400. 10.1139/f04-207 (2004).

[6] Kovach, R. P., Joyce, J. E., Echave, J. D., Lindberg, M. S. & Tallmon, D. A. Earlier migration timing, decreasing phenotypic variation, and biocomplexity in multiple salmonid species. *PLOS ONE*. **8**, e53807 (2013). 23326513 10.1371/journal.pone.0053807 PMC3542326

[7] Lehikoinen, E., Sparks, T. H. & Zalakevicius, M. Arrival and departure dates. In: *Advances in Ecological Research* 35 1–31 (Academic Press, 2004).

[8] Menzel, A. et al. European phenological response to climate change matches the warming pattern. *Glob Change Biol.* **12**, 1969–1976 (2006).

[9] Rubolini, D., Møller, A. P., Rainio, K. & Lehikoinen, E. Intraspecific consistency and geographic variability in temporal trends of spring migration phenology among European bird species. *Clim. Res.* **35**, 135–146 (2007).

[10] Saino, N. et al. Climate warming, ecological mismatch at arrival and population decline in migratory birds. *Proc. Biol. Sci.* **278**, 835–842 (2011). 20861045 10.1098/rspb.2010.1778 PMC3049050

[11] Visser, M. E. & Both, C. Shifts in phenology due to global climate change: the need for a yardstick. *Proc. R. Soc. B Biol. Sci.* 272, 2561–2569 (2005). 10.1098/rspb.2005.3356 PMC1559974 16321776

[12] Kelly, M. Adaptation to climate change through genetic accommodation and assimilation of plastic phenotypes. *Philos. Trans. R Soc. B Biol. Sci.* **374**, 20180176 (2019). 10.1098/rstb.2018.0176 PMC6365860 30966963

[13] Schlichting, C. D. & Wund, M. A. Phenotypic plasticity and epigenetic marking: An assessment of evidence for genetic accommodation. *Evolution* **68** (3), 656–672. 10.1111/evo.12348 (2014). 24410266 10.1111/evo.12348

[14] Garcia-Costoya, G., Williams, C. E., Faske, T. M., Moorman, J. D. & Logan, M. L. Evolutionary constraints mediate extinction risk under climate change. *Ecol. Lett.* **26**, 529–539 (2023). 36756845 10.1111/ele.14173

[15] Boyle, E. A., Li, Y. I. & Pritchard, J. K. An expanded view of complex traits: from polygenic to omnigenic. *Cell* **169**, 1177–1186 (2017). 28622505 10.1016/j.cell.2017.05.038 PMC5536862

[16] Turchin, M. C. et al. Evidence of widespread selection on standing variation in Europe at height-associated SNPs. *Nat. Genet.* **44**, 1015–1019 (2012). 22902787 10.1038/ng.2368 PMC3480734

[17] Toews, D. P. L., Taylor, S. A., Streby, H. M., Kramer, G. R. & Lovette, I. J. Selection on VPS13A linked to migration in a songbird. *Proc. Natl. Acad. Sci.* 116, 18272–18274 (2019). 10.1073/pnas.1909186116 PMC6744891 31451666

[18] Muñoz-Braceras, S., Tornero-Écija, A. R., Vincent, O. & Escalante, R. VPS13A is closely associated with mitochondria and is required for efficient lysosomal degradation. *Dis. Model. Mech.* **12**, dmm036681 (2019). 30709847 10.1242/dmm.036681 PMC6398486

[19] Yeshaw, W. M. et al. Human VPS13A is associated with multiple organelles and influences mitochondrial morphology and lipid droplet motility. *eLife* **8**, e43561 (2019). 30741634 10.7554/eLife.43561 PMC6389287

[20] Jenni-Eiermann, S., Jenni, L., Smith, S. & Costantini, D. Oxidative stress in endurance flight: An unconsidered factor in bird migration. *PLoS ONE*. **9**, e97650 (2014). 24830743 10.1371/journal.pone.0097650 PMC4022615

[21] Hess, J. E., Zendt, J. S., Matala, A. R. & Narum, S. R. Genetic basis of adult migration timing in anadromous steelhead discovered through multivariate association testing. *Proc. R. Soc. B Biol. Sci.* 283, 20153064 (2016). 10.1098/rspb.2015.3064 PMC4874702 27170720

[22] Prince, D. J. et al. The evolutionary basis of premature migration in Pacific salmon highlights the utility of genomics for informing conservation. *Sci. Adv.* **3**, e1603198 (2017). 28835916 10.1126/sciadv.1603198 PMC5559211

[23] Thompson, T. Q. et al. Anthropogenic habitat alteration leads to rapid loss of adaptive variation and restoration potential in wild salmon populations. *Proc. Natl. Acad. Sci.* **116**, 177–186 (2019). 30514813 10.1073/pnas.1811559115 PMC6320526

[24] Vera, L. M. et al. Circadian rhythm of preferred temperature in fish: Behavioural thermoregulation linked to daily photocycles in zebrafish and Nile tilapia. *J. Therm. Biol.* **113**, 103544 (2023). 37055103 10.1016/j.jtherbio.2023.103544

[25] Baker, H. K., Obedzinski, M., Grantham, T. E. & Carlson, S. M. Variation in salmon migration phenology bolsters population stability but is threatened by drought. *Ecol. Lett.* **28** (2), e70081. 10.1111/ele.70081 (2025). 39988798 10.1111/ele.70081 PMC11848020

[26] Herrera-R, G. A. et al. A synthesis of the diversity of freshwater fish migrations in the Amazon basin. *Fish. Fish.* **25**, 114–133 (2024).

[27] Moore, J. W., Yeakel, J. D., Peard, D., Lough, J. & Beere, M. Life-history diversity and its importance to population stability and persistence of a migratory fish: steelhead in two large North American watersheds. *J. Anim. Ecol.* **83**, 1035–1046 (2014). 24673479 10.1111/1365-2656.12212

[28] Tanaka, R., Hirashima, K., Kunishima, T., Uno, H. & Sato, T. Phenological diversity of freshwater migration can prolong assemblage-level migration period in amphidromous fishes in a temperate river system in Japan. *Ecol. Res.* **35**, 494–503 (2020).

[29] Vähä, J. P. et al. Temporally stable population-specific differences in run timing of one-sea-winter Atlantic salmon returning to a large river system. *Evol. Appl.* **4**, 39–53 (2011). 25567952 10.1111/j.1752-4571.2010.00131.x PMC3352515

[30] Glasauer, S. M. K. & Neuhauss, S. C. F. Whole-genome duplication in teleost fishes and its evolutionary consequences. *Mol. Genet. Genomics MGG*. **289**, 1045–1060 (2014). 25092473 10.1007/s00438-014-0889-2

[31] Alexandrou, M. A., Swartz, B. A., Matzke, N. J. & Oakley, T. H. Genome duplication and multiple evolutionary origins of complex migratory behavior in Salmonidae. *Mol. Phylogenet Evol.* **69**, 514–523 (2013). 23933489 10.1016/j.ympev.2013.07.026

[32] Lien, S. et al. The Atlantic salmon genome provides insights into rediploidization. *Nature* **533**, 200–205 (2016). 27088604 10.1038/nature17164 PMC8127823

[33] West, A. C. et al. Diversified regulation of circadian clock gene expression following whole genome duplication. *PLoS Genet.* **16**, e1009097 (2020). 33031398 10.1371/journal.pgen.1009097 PMC7575087

[34] O’Malley, K. G., Ford, M. J. & Hard, J. J. Clock polymorphism in Pacific salmon: evidence for variable selection along a latitudinal gradient. *Proc. R. Soc. B Biol. Sci.* 277, 3703–3714 (2010). 10.1098/rspb.2010.0762 PMC2992699 20610428

[35] Buhr, E. D. & Takahashi, J. S. Molecular components of the mammalian circadian clock. *Handb. Exp. Pharmacol.* 3–27. 10.1007/978-3-642-25950-0_1 (2013). 10.1007/978-3-642-25950-0_1 PMC3762864 23604473

[36] O’Malley, K. G. et al. Circadian clock gene (OtsClock1b) variation and time of ocean return in Atlantic salmon *Salmo salar*. *Fish. Manag. Ecol.* **21** (1), 82–87. 10.1111/fme.12058 (2014).

[37] Bradbury, I. R. et al. Range-wide genetic assignment confirms long-distance oceanic migration in Atlantic salmon over half a century. *ICES J. Mar. Sci.* **78**, 1434–1443 (2021).

[38] ICES. *Working Group on North Atlantic Salmon (WGNAS)*. 22499247 Bytes, 415 https://ices-library.figshare.com/articles/report/Working_Group_on_North_Atlantic_Salmon_WGNAS_/25730247/1 (2024). 10.17895/ICES.PUB.25730247.V1

[39] Lehnert, S. J. et al. Genomic signatures and correlates of widespread population declines in salmon. *Nat. Commun.* **10**, 2996 (2019). 31278264 10.1038/s41467-019-10972-w PMC6611788

[40] Otero, J. et al. Basin-scale phenology and effects of climate variability on global timing of initial seaward migration of Atlantic salmon (Salmo salar). *Glob. Change Biol.* **20** (1), 61–75. 10.1111/gcb.12363 (2014). 10.1111/gcb.12363 23966281

[41] Cauwelier, E., Gilbey, J., Sampayo, J., Stradmeyer, L. & Middlemas, S. J. Identification of a single genomic region associated with seasonal river return timing in adult Scottish Atlantic salmon (*Salmo salar*), using a genome-wide association study. *Can. J. Fish. Aquat. Sci.* **75** (9), 1427–1435. 10.1139/cjfas-2017-0293 (2018).

[42] Miettinen, A. et al. Temporal allele frequency changes in large-effect loci reveal potential fishing impacts on salmon life-history diversity. *Evol. Appl.* **17**, e13690 (2024). 38681510 10.1111/eva.13690 PMC11046039

[43] Pritchard, V. L. et al. Genomic signatures of fine-scale local selection in Atlantic salmon suggest involvement of sexual maturation, energy homeostasis and immune defence-related genes. *Mol. Ecol.* **27**, 2560–2575 (2018). 29691916 10.1111/mec.14705

[44] Beck, S. V. et al. Genomic basis and climate change vulnerability of migration timing in Atlantic Salmon (*Salmo salar*). *Evol. Appl.* **18**, e70148 (2025). 41019293 10.1111/eva.70148 PMC12474562

[45] Ayllon, F. et al. The vgll3 locus controls age at maturity in wild and domesticated Atlantic salmon (*Salmo salar L*.) males. *PLoS Genet.* **11** (11), e1005628. 10.1371/journal.pgen.1005628 (2015). 26551894 10.1371/journal.pgen.1005628 PMC4638356

[46] Barson, N. J. et al. Sex-dependent dominance at a single locus maintains variation in age at maturity in salmon. *Nature* **528**, 405–408 (2015). 26536110 10.1038/nature16062

[47] Sinclair-Waters, M. et al. Beyond large-effect loci: large-scale GWAS reveals a mixed large-effect and polygenic architecture for age at maturity of Atlantic salmon. *Genet. Sel. Evol.* **52**, 9 (2020). 32050893 10.1186/s12711-020-0529-8 PMC7017552

[48] Waters, C. D. et al. Heterogeneous genetic basis of age at maturity in salmonid fishes. *Mol. Ecol.* **30**, 1435–1456 (2021). 33527498 10.1111/mec.15822

[49] Willis, S. C. et al. Steelhead (*Oncorhynchus mykiss*) lineages and sexes show variable patterns of association of adult migration timing and age-at-maturity traits with two genomic regions. *Evol. Appl.* **13** (10), 2836–2856. 10.1111/eva.13088 (2020). 33294026 10.1111/eva.13088 PMC7691471

[50] Barry, P. D. et al. A major effect locus involved in migration timing is shared by pink and sockeye salmon. 03.30.587279 Preprint at 10.1101/2024.03.30.587279 (2024).

[51] Kess, T. et al. Variable parallelism in the genomic basis of age at maturity across spatial scales in Atlantic Salmon. *Ecol. Evol.* **14**, e11068 (2024). 38584771 10.1002/ece3.11068 PMC10995719

[52] Andrews, S. & FastQC: A quality control tool for high throughput sequence data. (2010).

[53] Martin, M. Cutadapt removes adapter sequences from high-throughput sequencing reads. *EMBnet J.* **17**, 10 (2011).

[54] Li, H. Aligning sequence reads, clone sequences and assembly contigs with BWA-MEM. Preprint at http://arxiv.org/abs/1303.3997 (2013).

[55] DePristo, M. A. et al. A framework for variation discovery and genotyping using next-generation DNA sequencing data. *Nat. Genet.* **43**, 491–498 (2011). 21478889 10.1038/ng.806 PMC3083463

[56] Korneliussen, T. S., Albrechtsen, A. & Nielsen, R. ANGSD: Analysis of next generation sequencing data. *BMC Bioinform.* **15**, 356 (2014). 10.1186/s12859-014-0356-4 PMC4248462 25420514

[57] Danecek, P. et al. The variant call format and VCFtools. *Bioinformatics* **27**, 2156–2158 (2011). 21653522 10.1093/bioinformatics/btr330 PMC3137218

[58] Browning, S. R. & Browning, B. L. Rapid and accurate haplotype phasing and missing-data inference for whole-genome association studies by use of localized haplotype clustering. *Am. J. Hum. Genet.* **81** (5), 1084–1097. 10.1086/521987 (2007). 17924348 10.1086/521987 PMC2265661

[59] Purcell, S. et al. PLINK: a tool set for whole-genome association and population-based linkage analyses. *Am. J. Hum. Genet.* **81**, 559–575 (2007). 17701901 10.1086/519795 PMC1950838

[60] Jackson, D. A. Stopping rules in principal components analysis: a comparison of heuristical and statistical approaches. *Ecology* **74**, 2204–2214 (1993).

[61] Marees, A. T. et al. A tutorial on conducting genome-wide association studies: Quality control and statistical analysis. *Int. J. Methods Psychiatr Res.* **27**, e1608 (2018). 29484742 10.1002/mpr.1608 PMC6001694

[62] Benjamini, Y. & Hochberg, Y. Controlling the false discovery rate: A practical and powerful approach to multiple testing. *J. Roy. Stat. Soc.: Ser. B (Methodol.)*. **57** (1), 289–300. 10.1111/j.2517-6161.1995.tb02031.x (1995).

[63] Quinlan, A. R. & Hall, I. M. BEDTools: a flexible suite of utilities for comparing genomic features. *Bioinforma Oxf. Engl.* **26**, 841–842 (2010). 10.1093/bioinformatics/btq033 PMC2832824 20110278

[64] Ge, S. X., Jung, D. & Yao, R. ShinyGO: a graphical gene-set enrichment tool for animals and plants. *Bioinformatics* **36**, 2628–2629 (2020). 31882993 10.1093/bioinformatics/btz931 PMC7178415

[65] Kanehisa, M., Furumichi, M., Tanabe, M., Sato, Y. & Morishima, K. KEGG: new perspectives on genomes, pathways, diseases and drugs. *Nucleic Acids Res.* **45**, D353–D361 (2017). 27899662 10.1093/nar/gkw1092 PMC5210567

[66] Ashburner, M. et al. Gene ontology: tool for the unification of biology. *Nat. Genet.* **25**, 25–29 (2000). 10802651 10.1038/75556 PMC3037419

[67] Lameris, T. K. et al. Arctic geese tune migration to a warming climate but still suffer from a phenological mismatch. *Curr. Biol.* **28**, 2467–2473 (2018). 30033332 10.1016/j.cub.2018.05.077

[68] Reed, T. E., Jenouvrier, S. & Visser, M. E. Phenological mismatch strongly affects individual fitness but not population demography in a woodland passerine. *J. Anim. Ecol.* **82**, 131–144 (2013). 22862682 10.1111/j.1365-2656.2012.02020.x

[69] Visser, M. E., Holleman, L. J. M. & Gienapp, P. Shifts in caterpillar biomass phenology due to climate change and its impact on the breeding biology of an insectivorous bird. *Oecologia* **147**, 164–172 (2006). 16328547 10.1007/s00442-005-0299-6

[70] Charmantier, A. & Gienapp, P. Climate change and timing of avian breeding and migration: evolutionary versus plastic changes. *Evol. Appl.* **7**, 15–28 (2014). 24454545 10.1111/eva.12126 PMC3894895

[71] de Greef, E., Suh, A., Thorstensen, M. J., Delmore, K. E. & Fraser, K. C. Genomic architecture of migration timing in a long-distance migratory songbird. *Sci. Rep.* **13**, 2437 (2023). 36765096 10.1038/s41598-023-29470-7 PMC9918537

[72] Conte, I. et al. Proper differentiation of photoreceptors and amacrine cells depends on a regulatory loop between NeuroD and Six6. *Development* **137**, 2307–2317 (2010). 20534668 10.1242/dev.045294

[73] Iglesias, A. I. et al. Exome sequencing and functional analyses suggest that SIX6 is a gene involved in an altered proliferation–differentiation balance early in life and optic nerve degeneration at old age. *Hum. Mol. Genet.* **23**, 1320–1332 (2014). 24150847 10.1093/hmg/ddt522

[74] Ulmer Carnes, M. et al. Discovery and functional annotation of SIX6 variants in primary open-angle glaucoma. *PLoS Genet.* **10**, e1004372 (2014). 24875647 10.1371/journal.pgen.1004372 PMC4038608

[75] Clark, D. D. et al. Aberrant development of the suprachiasmatic nucleus and circadian rhythms in mice lacking the homeodomain protein Six6. *J. Biol. Rhythms*. **28**, 15–25 (2013). 23382588 10.1177/0748730412468084 PMC3586279

[76] Landgraf, D., Koch, C. E. & Oster, H. Embryonic development of circadian clocks in the mammalian suprachiasmatic nuclei. *Front Neuroanat* **8**, (2014). 10.3389/fnana.2014.00143 PMC4249487 25520627

[77] Larder, R., Clark, D. D., Miller, N. L. G. & Mellon, P. L. Hypothalamic dysregulation and infertility in mice lacking the homeodomain protein Six6. *J. Neurosci.* **31**, 426–438 (2011). 21228153 10.1523/JNEUROSCI.1688-10.2011 PMC3103738

[78] Pandolfi, E. C., Tonsfeldt, K. J., Hoffmann, H. M. & Mellon, P. L. Deletion of the homeodomain protein Six6 from GnRH neurons decreases GnRH gene expression, resulting in infertility. *Endocrinology* **160**, 2151–2164 (2019). 31211355 10.1210/en.2019-00113 PMC6821215

[79] Kjærner-Semb, E. et al. Comparison of anadromous and landlocked Atlantic salmon genomes reveals signatures of parallel and relaxed selection across the Northern Hemisphere. *Evol. Appl.* **14**, 446–461 (2020). 33664787 10.1111/eva.13129 PMC7896726

[80] Husak, J. F. & Lailvaux, S. P. Conserved and convergent mechanisms underlying performance–life-history trade-offs. *J. Exp. Biol.* **225**, jeb243351 (2022). 35119073 10.1242/jeb.243351

[81] Quinn, T. P., McGinnity, P. & Reed, T. E. The paradox of premature migration by adult anadromous salmonid fishes: patterns and hypotheses. *Can. J. Fish. Aquat. Sci.* **73**, 1015–1030 (2016).

[82] Dingle, H. Animal migration: is there a common migratory syndrome? *J. Ornithol.* **147**, 212–220 (2006).

[83] Figge, F. Bio-folio: applying portfolio theory to biodiversity. *Biodivers. Conserv.* **13**, 827–849 (2004).

[84] Mangiavacchi, A., Morelli, G. & Orlando, V. Behind the scenes: How RNA orchestrates the epigenetic regulation of gene expression. *Front Cell. Dev. Biol* **11**, (2023). 10.3389/fcell.2023.1123975 PMC9905133 36760365

[85] Tak, Y. G. & Farnham, P. J. Making sense of GWAS: using epigenomics and genome engineering to understand the functional relevance of SNPs in non-coding regions of the human genome. *Epigenetics Chromatin*. **8**, 57 (2015). 26719772 10.1186/s13072-015-0050-4 PMC4696349

[86] Feil, R. & Fraga, M. F. Epigenetics and the environment: emerging patterns and implications. *Nat. Rev. Genet.* **13**, 97–109 (2012). 22215131 10.1038/nrg3142

[87] Kaikkonen, M. U., Lam, M. T. Y. & Glass, C. K. Non-coding RNAs as regulators of gene expression and epigenetics. *Cardiovasc. Res.* **90**, 430–440 (2011). 21558279 10.1093/cvr/cvr097 PMC3096308

[88] Kolovos, P., Knoch, T. A., Grosveld, F. G., Cook, P. R. & Papantonis, A. Enhancers and silencers: an integrated and simple model for their function. *Epigenetics Chromatin*. **5**, 1 (2012). 22230046 10.1186/1756-8935-5-1 PMC3281776

[89] Sollars, V. et al. Evidence for an epigenetic mechanism by which Hsp90 acts as a capacitor for morphological evolution. *Nat. Genet.* **33**, 70–74 (2003). 12483213 10.1038/ng1067

[90] Angers, B., Castonguay, E. & Massicotte, R. Environmentally induced phenotypes and DNA methylation: how to deal with unpredictable conditions until the next generation and after. *Mol. Ecol.* **19**, 1283–1295 (2010). 20298470 10.1111/j.1365-294X.2010.04580.x

[91] Leung, C., Breton, S. & Angers, B. Facing environmental predictability with different sources of epigenetic variation. *Ecol. Evol.* **6**, 5234–5245 (2016). 27551379 10.1002/ece3.2283 PMC4984500

[92] Prather, R. M. et al. Current and lagged climate affects phenology across diverse taxonomic groups. *Proc. R. Soc. B Biol. Sci.* 290, 20222181 (2023). 10.1098/rspb.2022.2181 PMC9832555 36629105

[93] Venney, C. J. et al. Genome-wide DNA methylation predicts environmentally driven life history variation in a marine fish. *Evolution* **77**, 186–198 (2023). 36622671 10.1093/evolut/qpac028

