# Supplementary material for: A large-effect locus underlies migration timing in North American Atlantic salmon (Salmo salar)
Source: Sci Rep. 2026 Mar 2;16:11543. doi: 10.1038/s41598-026-42281-w (PMC13057212; doi:10.1038/s41598-026-42281-w)
Supplement: Supplementary file 1 — Supplementary Material 1 [file 41598_2026_42281_MOESM1_ESM.docx]

**Table S1** All associations, including non-coding regions, with adult return timing in seven populations of North American Atlantic salmon (*Salmo salar*) using lc-WGS and corrected for population structure (K = 7), age-at-maturity and sex. Ordered according to significance according to the Benjamini-Hochberg false discovery rate correction (FDR_BH).

| **Chromosome** | **BP** | **FDR_BH** | **SNP** |
| --- | --- | --- | --- |
| ssa17 | 63670009 | 0.0001406 | NA6644450_NC_059458.1_63670009 |
| ssa17 | 63674808 | 0.0001406 | NA6644460_NC_059458.1_63674808 |
| ssa17 | 63677797 | 0.0001406 | NA6644474_NC_059458.1_63677797 |
| ssa17 | 63665677 | 0.0004794 | NA6644418_NC_059458.1_63665677 |
| ssa17 | 63666534 | 0.0004794 | NA6644421_NC_059458.1_63666534 |
| ssa17 | 63638643 | 0.001202 | NA6644198_NC_059458.1_63638643 |
| ssa17 | 63719955 | 0.001294 | NA6644706_NC_059458.1_63719955 |
| ssa17 | 63645736 | 0.001896 | NA6644311_NC_059458.1_63645736 |
| ssa17 | 63693716 | 0.001896 | NA6644617_NC_059458.1_63693716 |
| ssa26 | 19720213 | 0.004331 | NA9005918_NC_059467.1_19720213 |
| ssa17 | 63659914 | 0.005891 | NA6644387_NC_059458.1_63659914 |
| ssa17 | 63669912 | 0.005891 | NA6644447_NC_059458.1_63669912 |
| ssa17 | 63779053 | 0.005891 | NA6644958_NC_059458.1_63779053 |
| ssa09 | 149982848 | 0.006664 | NA3485901_NC_059450.1_149982848 |
| ssa17 | 7686510 | 0.006664 | NA6457463_NC_059458.1_7686510 |
| ssa17 | 7687786 | 0.006664 | NA6457466_NC_059458.1_7687786 |
| ssa17 | 7688468 | 0.006664 | NA6457468_NC_059458.1_7688468 |
| ssa17 | 63641852 | 0.006664 | NA6644209_NC_059458.1_63641852 |
| ssa17 | 63666957 | 0.006664 | NA6644426_NC_059458.1_63666957 |
| ssa17 | 63719798 | 0.006664 | NA6644705_NC_059458.1_63719798 |
| ssa17 | 63662368 | 0.006891 | NA6644395_NC_059458.1_63662368 |
| ssa16 | 89373349 | 0.007347 | NA6427375_NC_059457.1_89373349 |
| ssa17 | 63777196 | 0.007389 | NA6644947_NC_059458.1_63777196 |
| ssa09 | 132681362 | 0.007541 | NA3414658_NC_059450.1_132681362 |
| ssa09 | 132681456 | 0.007541 | NA3414659_NC_059450.1_132681456 |
| ssa12 | 25774009 | 0.007541 | NA4555891_NC_059453.1_25774009 |
| ssa17 | 63573783 | 0.007573 | NA6643819_NC_059458.1_63573783 |
| ssa09 | 149982847 | 0.008266 | NA3485900_NC_059450.1_149982847 |
| ssa17 | 63637324 | 0.008266 | NA6644191_NC_059458.1_63637324 |
| ssa17 | 63659529 | 0.008266 | NA6644386_NC_059458.1_63659529 |
| ssa17 | 63663566 | 0.008266 | NA6644406_NC_059458.1_63663566 |
| ssa17 | 63664079 | 0.008266 | NA6644407_NC_059458.1_63664079 |
| ssa17 | 63680439 | 0.008266 | NA6644522_NC_059458.1_63680439 |
| ssa17 | 63696615 | 0.008266 | NA6644635_NC_059458.1_63696615 |
| ssa17 | 63527371 | 0.008297 | NA6643679_NC_059458.1_63527371 |
| ssa17 | 63679503 | 0.008297 | NA6644513_NC_059458.1_63679503 |
| ssa01 | 140861645 | 0.008955 | NA530059_NC_059442.1_140861645 |
| ssa17 | 63641554 | 0.009043 | NA6644208_NC_059458.1_63641554 |
| ssa09 | 1277607 | 0.009475 | NA2958089_NC_059450.1_1277607 |
| ssa09 | 132685519 | 0.009475 | NA3414671_NC_059450.1_132685519 |
| ssa12 | 25773963 | 0.009475 | NA4555890_NC_059453.1_25773963 |
| ssa14 | 78989614 | 0.009475 | NA5560767_NC_059455.1_78989614 |
| ssa17 | 63657664 | 0.009475 | NA6644378_NC_059458.1_63657664 |
| ssa17 | 63664676 | 0.009475 | NA6644410_NC_059458.1_63664676 |
| ssa17 | 63676065 | 0.009475 | NA6644462_NC_059458.1_63676065 |
| ssa17 | 63676152 | 0.009475 | NA6644463_NC_059458.1_63676152 |
| ssa17 | 63703696 | 0.009475 | NA6644652_NC_059458.1_63703696 |
| ssa17 | 63704186 | 0.009475 | NA6644657_NC_059458.1_63704186 |
| ssa17 | 63778720 | 0.009475 | NA6644956_NC_059458.1_63778720 |
| ssa17 | 63637610 | 0.00954 | NA6644193_NC_059458.1_63637610 |
| ssa17 | 7689065 | 0.009784 | NA6457495_NC_059458.1_7689065 |
| ssa15 | 64737257 | 0.009883 | NA5948796_NC_059456.1_64737257 |
| ssa17 | 63678418 | 0.009883 | NA6644475_NC_059458.1_63678418 |
| ssa17 | 63687303 | 0.009883 | NA6644551_NC_059458.1_63687303 |
| ssa17 | 63687911 | 0.009883 | NA6644553_NC_059458.1_63687911 |
| ssa17 | 63700162 | 0.009883 | NA6644643_NC_059458.1_63700162 |
| ssa17 | 63658755 | 0.009884 | NA6644381_NC_059458.1_63658755 |
| ssa17 | 63676249 | 0.009884 | NA6644464_NC_059458.1_63676249 |
| ssa17 | 63661959 | 0.01179 | NA6644394_NC_059458.1_63661959 |
| ssa17 | 63684692 | 0.01181 | NA6644535_NC_059458.1_63684692 |
| ssa17 | 63684912 | 0.01181 | NA6644536_NC_059458.1_63684912 |
| ssa17 | 63684913 | 0.01181 | NA6644537_NC_059458.1_63684913 |
| ssa17 | 63687754 | 0.01181 | NA6644552_NC_059458.1_63687754 |
| ssa17 | 64737682 | 0.01181 | NA6649709_NC_059458.1_64737682 |
| ssa25 | 22695906 | 0.01181 | NA8789586_NC_059466.1_22695906 |
| ssa25 | 22696536 | 0.01181 | NA8789587_NC_059466.1_22696536 |
| ssa09 | 149982857 | 0.01184 | NA3485902_NC_059450.1_149982857 |
| ssa07 | 14731264 | 0.01238 | NA2641300_NC_059448.1_14731264 |
| ssa17 | 63638201 | 0.01238 | NA6644196_NC_059458.1_63638201 |
| ssa17 | 63690903 | 0.01238 | NA6644604_NC_059458.1_63690903 |
| ssa01 | 140928418 | 0.01308 | NA530238_NC_059442.1_140928418 |
| ssa09 | 152196151 | 0.01308 | NA3498079_NC_059450.1_152196151 |
| ssa15 | 64731197 | 0.01308 | NA5948780_NC_059456.1_64731197 |
| ssa15 | 64734747 | 0.01308 | NA5948791_NC_059456.1_64734747 |
| ssa15 | 64737809 | 0.01308 | NA5948817_NC_059456.1_64737809 |
| ssa17 | 63557681 | 0.01308 | NA6643790_NC_059458.1_63557681 |
| ssa17 | 63570778 | 0.01308 | NA6643810_NC_059458.1_63570778 |
| ssa17 | 63655242 | 0.01308 | NA6644363_NC_059458.1_63655242 |
| ssa17 | 63666898 | 0.01308 | NA6644424_NC_059458.1_63666898 |
| ssa12 | 25773380 | 0.01455 | NA4555889_NC_059453.1_25773380 |
| ssa17 | 63657066 | 0.0152 | NA6644372_NC_059458.1_63657066 |
| ssa09 | 1284320 | 0.01547 | NA2958123_NC_059450.1_1284320 |
| ssa09 | 1284464 | 0.01547 | NA2958124_NC_059450.1_1284464 |
| ssa15 | 64727986 | 0.01547 | NA5948775_NC_059456.1_64727986 |
| ssa15 | 64728472 | 0.01547 | NA5948779_NC_059456.1_64728472 |
| ssa17 | 63535438 | 0.01547 | NA6643708_NC_059458.1_63535438 |
| ssa17 | 63690045 | 0.01547 | NA6644597_NC_059458.1_63690045 |
| ssa24 | 8838209 | 0.01547 | NA8528726_NC_059465.1_8838209 |
| ssa17 | 63537932 | 0.01609 | NA6643736_NC_059458.1_63537932 |
| ssa20 | 66215494 | 0.01712 | NA7613190_NC_059461.1_66215494 |
| ssa17 | 63686662 | 0.01758 | NA6644548_NC_059458.1_63686662 |
| ssa09 | 1234845 | 0.01761 | NA2957853_NC_059450.1_1234845 |
| ssa17 | 63684482 | 0.01845 | NA6644534_NC_059458.1_63684482 |
| ssa15 | 64718579 | 0.01951 | NA5948754_NC_059456.1_64718579 |
| ssa15 | 64727546 | 0.01951 | NA5948774_NC_059456.1_64727546 |
| ssa25 | 22570987 | 0.01951 | NA8789067_NC_059466.1_22570987 |
| ssa17 | 63676664 | 0.01982 | NA6644466_NC_059458.1_63676664 |
| ssa17 | 63666218 | 0.02103 | NA6644420_NC_059458.1_63666218 |
| ssa17 | 63690152 | 0.02109 | NA6644600_NC_059458.1_63690152 |
| ssa02 | 76653146 | 0.02116 | NA954813_NC_059443.1_76653146 |
| ssa17 | 63534303 | 0.02116 | NA6643706_NC_059458.1_63534303 |
| ssa17 | 63687978 | 0.02224 | NA6644554_NC_059458.1_63687978 |
| ssa09 | 152196149 | 0.02282 | NA3498078_NC_059450.1_152196149 |
| ssa17 | 63685417 | 0.02342 | NA6644541_NC_059458.1_63685417 |
| ssa17 | 63778939 | 0.02342 | NA6644957_NC_059458.1_63778939 |
| ssa01 | 140768182 | 0.02426 | NA529861_NC_059442.1_140768182 |
| ssa17 | 64221053 | 0.02501 | NA6647485_NC_059458.1_64221053 |
| ssa17 | 64221054 | 0.02501 | NA6647486_NC_059458.1_64221054 |
| ssa01 | 140725011 | 0.02567 | NA529780_NC_059442.1_140725011 |
| ssa09 | 1234027 | 0.02567 | NA2957840_NC_059450.1_1234027 |
| ssa09 | 1283229 | 0.02567 | NA2958118_NC_059450.1_1283229 |
| ssa09 | 1283655 | 0.02567 | NA2958121_NC_059450.1_1283655 |
| ssa09 | 78633597 | 0.02567 | NA3230928_NC_059450.1_78633597 |
| ssa15 | 64724436 | 0.02567 | NA5948768_NC_059456.1_64724436 |
| ssa17 | 63703050 | 0.02705 | NA6644651_NC_059458.1_63703050 |
| ssa01 | 140765116 | 0.02706 | NA529856_NC_059442.1_140765116 |
| ssa17 | 63665389 | 0.02706 | NA6644417_NC_059458.1_63665389 |
| ssa01 | 140730915 | 0.02756 | NA529791_NC_059442.1_140730915 |
| ssa15 | 64723913 | 0.02756 | NA5948767_NC_059456.1_64723913 |
| ssa09 | 1288851 | 0.02785 | NA2958140_NC_059450.1_1288851 |
| ssa07 | 51965584 | 0.02837 | NA2795230_NC_059448.1_51965584 |
| ssa09 | 78632209 | 0.03046 | NA3230922_NC_059450.1_78632209 |
| ssa09 | 132652875 | 0.03078 | NA3414538_NC_059450.1_132652875 |
| ssa01 | 35067470 | 0.03174 | NA159685_NC_059442.1_35067470 |
| ssa01 | 140782277 | 0.03286 | NA529895_NC_059442.1_140782277 |
| ssa16 | 4115755 | 0.03286 | NA6140278_NC_059457.1_4115755 |
| ssa17 | 63686566 | 0.03359 | NA6644547_NC_059458.1_63686566 |
| ssa01 | 140767540 | 0.0336 | NA529857_NC_059442.1_140767540 |
| ssa17 | 63650924 | 0.0336 | NA6644329_NC_059458.1_63650924 |
| ssa01 | 140742351 | 0.03412 | NA529823_NC_059442.1_140742351 |
| ssa17 | 63666967 | 0.03412 | NA6644427_NC_059458.1_63666967 |
| ssa01 | 118613520 | 0.03419 | NA458621_NC_059442.1_118613520 |
| ssa01 | 118613522 | 0.03419 | NA458622_NC_059442.1_118613522 |
| ssa01 | 140922794 | 0.03419 | NA530223_NC_059442.1_140922794 |
| ssa09 | 78763489 | 0.03419 | NA3231377_NC_059450.1_78763489 |
| ssa15 | 64731969 | 0.03419 | NA5948786_NC_059456.1_64731969 |
| ssa17 | 63647733 | 0.03419 | NA6644324_NC_059458.1_63647733 |
| ssa17 | 63649982 | 0.03419 | NA6644327_NC_059458.1_63649982 |
| ssa17 | 63690855 | 0.03471 | NA6644603_NC_059458.1_63690855 |
| ssa15 | 64717461 | 0.03644 | NA5948751_NC_059456.1_64717461 |
| ssa15 | 64717614 | 0.03673 | NA5948752_NC_059456.1_64717614 |
| ssa07 | 51965581 | 0.03684 | NA2795228_NC_059448.1_51965581 |
| ssa21 | 23570970 | 0.03684 | NA7842975_NC_059462.1_23570970 |
| ssa17 | 63647511 | 0.03747 | NA6644317_NC_059458.1_63647511 |
| ssa17 | 63654699 | 0.03747 | NA6644362_NC_059458.1_63654699 |
| ssa01 | 140832594 | 0.03841 | NA529996_NC_059442.1_140832594 |
| ssa11 | 45560553 | 0.03845 | NA4180613_NC_059452.1_45560553 |
| ssa18 | 55657076 | 0.03845 | NA6927187_NC_059459.1_55657076 |
| ssa18 | 55657077 | 0.03845 | NA6927188_NC_059459.1_55657077 |
| ssa18 | 55657081 | 0.03845 | NA6927189_NC_059459.1_55657081 |
| ssa18 | 55657083 | 0.03845 | NA6927190_NC_059459.1_55657083 |
| ssa21 | 23570971 | 0.03845 | NA7842976_NC_059462.1_23570971 |
| ssa25 | 22686987 | 0.03845 | NA8789555_NC_059466.1_22686987 |
| ssa25 | 22690124 | 0.03845 | NA8789563_NC_059466.1_22690124 |
| ssa25 | 22690148 | 0.03845 | NA8789564_NC_059466.1_22690148 |
| ssa25 | 22695536 | 0.03845 | NA8789585_NC_059466.1_22695536 |
| ssa25 | 22696591 | 0.03845 | NA8789588_NC_059466.1_22696591 |
| ssa25 | 22697047 | 0.03845 | NA8789591_NC_059466.1_22697047 |
| ssa25 | 22708605 | 0.03845 | NA8789646_NC_059466.1_22708605 |
| ssa17 | 63643878 | 0.03911 | NA6644296_NC_059458.1_63643878 |
| ssa01 | 140935182 | 0.0392 | NA530254_NC_059442.1_140935182 |
| ssa15 | 64731206 | 0.03957 | NA5948781_NC_059456.1_64731206 |
| ssa17 | 63646680 | 0.04007 | NA6644315_NC_059458.1_63646680 |
| ssa28 | 36637557 | 0.04021 | NA9556155_NC_059469.1_36637557 |
| ssa10 | 55608902 | 0.04027 | NA3760124_NC_059451.1_55608902 |
| ssa25 | 22570991 | 0.04027 | NA8789068_NC_059466.1_22570991 |
| ssa17 | 63645191 | 0.04035 | NA6644308_NC_059458.1_63645191 |
| ssa17 | 63640581 | 0.04152 | NA6644201_NC_059458.1_63640581 |
| ssa12 | 25770288 | 0.04383 | NA4555876_NC_059453.1_25770288 |
| ssa15 | 64721288 | 0.04397 | NA5948758_NC_059456.1_64721288 |
| ssa15 | 64735882 | 0.04428 | NA5948794_NC_059456.1_64735882 |
| ssa17 | 63641325 | 0.04459 | NA6644203_NC_059458.1_63641325 |
| ssa01 | 140745756 | 0.04554 | NA529830_NC_059442.1_140745756 |
| ssa09 | 78642797 | 0.04554 | NA3230954_NC_059450.1_78642797 |
| ssa11 | 45243145 | 0.04669 | NA4179703_NC_059452.1_45243145 |
| ssa01 | 140770192 | 0.04743 | NA529864_NC_059442.1_140770192 |
| ssa09 | 1234107 | 0.04743 | NA2957842_NC_059450.1_1234107 |
| ssa09 | 1396068 | 0.04743 | NA2958616_NC_059450.1_1396068 |
| ssa15 | 64735973 | 0.04743 | NA5948795_NC_059456.1_64735973 |
| ssa17 | 63533446 | 0.0475 | NA6643702_NC_059458.1_63533446 |
| ssa12 | 88962886 | 0.04795 | NA4761817_NC_059453.1_88962886 |
| ssa17 | 63653678 | 0.04804 | NA6644358_NC_059458.1_63653678 |
| ssa17 | 63780270 | 0.04857 | NA6644970_NC_059458.1_63780270 |
| ssa12 | 25770838 | 0.04876 | NA4555880_NC_059453.1_25770838 |
| ssa12 | 25771178 | 0.04876 | NA4555881_NC_059453.1_25771178 |
| ssa01 | 140834761 | 0.0498 | NA530002_NC_059442.1_140834761 |
| ssa02 | 3341706 | 0.04988 | NA682678_NC_059443.1_3341706 |
| ssa09 | 78725028 | 0.04988 | NA3231256_NC_059450.1_78725028 |
| ssa12 | 90579679 | 0.04988 | NA4768409_NC_059453.1_90579679 |
| ssa17 | 63529628 | 0.04988 | NA6643683_NC_059458.1_63529628 |
| ssa17 | 63662556 | 0.04988 | NA6644396_NC_059458.1_63662556 |
| ssa17 | 63668172 | 0.04988 | NA6644438_NC_059458.1_63668172 |
| ssa17 | 63668173 | 0.04988 | NA6644439_NC_059458.1_63668173 |

**Table S2** Overlapping-gene associations with adult return timing in seven populations of North American Atlantic salmon (*Salmo salar*) using lc-WGS and corrected for population structure (K = 7), age-at-maturity and sex. Ordered according to significance according to the Benjamini-Hochberg false discovery rate correction (FDR_BH).

| **Chromosome** | **Position** | **gene** | **FDR_BH** | **SNP** |
| --- | --- | --- | --- | --- |
| ssa17 | 63638643 | ppfia2 | 0.001202 | NA6644198_NC_059458.1_63638643 |
| ssa17 | 63719955 | golgb1 | 0.001294 | NA6644706_NC_059458.1_63719955 |
| ssa17 | 63779053 | lhfpl3 | 0.005891 | NA6644958_NC_059458.1_63779053 |
| ssa17 | 7686510 | LOC106575122 | 0.006664 | NA6457463_NC_059458.1_7686510 |
| ssa17 | 7687786 | LOC106575122 | 0.006664 | NA6457466_NC_059458.1_7687786 |
| ssa17 | 7688468 | LOC106575122 | 0.006664 | NA6457468_NC_059458.1_7688468 |
| ssa17 | 63641852 | ppfia2 | 0.006664 | NA6644209_NC_059458.1_63641852 |
| ssa17 | 63719798 | golgb1 | 0.006664 | NA6644705_NC_059458.1_63719798 |
| ssa16 | 89373349 | LOC106574799 | 0.007347 | NA6427375_NC_059457.1_89373349 |
| ssa17 | 63777196 | lhfpl3 | 0.007389 | NA6644947_NC_059458.1_63777196 |
| ssa09 | 132681362 | cadm1b | 0.007541 | NA3414658_NC_059450.1_132681362 |
| ssa09 | 132681456 | cadm1b | 0.007541 | NA3414659_NC_059450.1_132681456 |
| ssa17 | 63573783 | ppfia2 | 0.007573 | NA6643819_NC_059458.1_63573783 |
| ssa17 | 63637324 | ppfia2 | 0.008266 | NA6644191_NC_059458.1_63637324 |
| ssa17 | 63527371 | ppfia2 | 0.008297 | NA6643679_NC_059458.1_63527371 |
| ssa17 | 63641554 | ppfia2 | 0.009043 | NA6644208_NC_059458.1_63641554 |
| ssa09 | 1277607 | LOC106610641 | 0.009475 | NA2958089_NC_059450.1_1277607 |
| ssa09 | 132685519 | cadm1b | 0.009475 | NA3414671_NC_059450.1_132685519 |
| ssa17 | 63703696 | LOC106564527 | 0.009475 | NA6644652_NC_059458.1_63703696 |
| ssa17 | 63704186 | LOC106564527 | 0.009475 | NA6644657_NC_059458.1_63704186 |
| ssa17 | 63778720 | lhfpl3 | 0.009475 | NA6644956_NC_059458.1_63778720 |
| ssa17 | 63637610 | ppfia2 | 0.00954 | NA6644193_NC_059458.1_63637610 |
| ssa17 | 7689065 | LOC106575122 | 0.009784 | NA6457495_NC_059458.1_7689065 |
| ssa15 | 64737257 | LOC106571912 | 0.009883 | NA5948796_NC_059456.1_64737257 |
| ssa17 | 63700162 | llph | 0.009883 | NA6644643_NC_059458.1_63700162 |
| ssa17 | 64737682 | chrm2a | 0.01181 | NA6649709_NC_059458.1_64737682 |
| ssa07 | 14731264 | LOC106608688 | 0.01238 | NA2641300_NC_059448.1_14731264 |
| ssa17 | 63638201 | ppfia2 | 0.01238 | NA6644196_NC_059458.1_63638201 |
| ssa09 | 152196151 | kcnab1a | 0.01308 | NA3498079_NC_059450.1_152196151 |
| ssa15 | 64731197 | LOC106571912 | 0.01308 | NA5948780_NC_059456.1_64731197 |
| ssa15 | 64734747 | LOC106571912 | 0.01308 | NA5948791_NC_059456.1_64734747 |
| ssa15 | 64737809 | LOC106571912 | 0.01308 | NA5948817_NC_059456.1_64737809 |
| ssa17 | 63557681 | ppfia2 | 0.01308 | NA6643790_NC_059458.1_63557681 |
| ssa17 | 63570778 | ppfia2 | 0.01308 | NA6643810_NC_059458.1_63570778 |
| ssa09 | 1284320 | LOC106610641 | 0.01547 | NA2958123_NC_059450.1_1284320 |
| ssa09 | 1284464 | LOC106610641 | 0.01547 | NA2958124_NC_059450.1_1284464 |
| ssa15 | 64727986 | LOC106571912 | 0.01547 | NA5948775_NC_059456.1_64727986 |
| ssa15 | 64728472 | LOC106571912 | 0.01547 | NA5948779_NC_059456.1_64728472 |
| ssa17 | 63535438 | ppfia2 | 0.01547 | NA6643708_NC_059458.1_63535438 |
| ssa24 | 8838209 | araf | 0.01547 | NA8528726_NC_059465.1_8838209 |
| ssa17 | 63537932 | ppfia2 | 0.01609 | NA6643736_NC_059458.1_63537932 |
| ssa09 | 1234845 | bahd1 | 0.01761 | NA2957853_NC_059450.1_1234845 |
| ssa15 | 64718579 | LOC106571912 | 0.01951 | NA5948754_NC_059456.1_64718579 |
| ssa15 | 64727546 | LOC106571912 | 0.01951 | NA5948774_NC_059456.1_64727546 |
| ssa25 | 22570987 | sema5ba | 0.01951 | NA8789067_NC_059466.1_22570987 |
| ssa02 | 76653146 | LOC106586284 | 0.02116 | NA954813_NC_059443.1_76653146 |
| ssa17 | 63534303 | ppfia2 | 0.02116 | NA6643706_NC_059458.1_63534303 |
| ssa09 | 152196149 | kcnab1a | 0.02282 | NA3498078_NC_059450.1_152196149 |
| ssa17 | 63778939 | lhfpl3 | 0.02342 | NA6644957_NC_059458.1_63778939 |
| ssa01 | 140768182 | LOC106565903 | 0.02426 | NA529861_NC_059442.1_140768182 |
| ssa17 | 64221053 | LOC106576364 | 0.02501 | NA6647485_NC_059458.1_64221053 |
| ssa17 | 64221054 | LOC106576364 | 0.02501 | NA6647486_NC_059458.1_64221054 |
| ssa01 | 140725011 | LOC106565903 | 0.02567 | NA529780_NC_059442.1_140725011 |
| ssa09 | 1234027 | bahd1 | 0.02567 | NA2957840_NC_059450.1_1234027 |
| ssa09 | 1283229 | LOC106610641 | 0.02567 | NA2958118_NC_059450.1_1283229 |
| ssa09 | 1283655 | LOC106610641 | 0.02567 | NA2958121_NC_059450.1_1283655 |
| ssa09 | 78633597 | si:zfos-2326c3.2 | 0.02567 | NA3230928_NC_059450.1_78633597 |
| ssa15 | 64724436 | LOC106571912 | 0.02567 | NA5948768_NC_059456.1_64724436 |
| ssa17 | 63703050 | LOC106564527 | 0.02705 | NA6644651_NC_059458.1_63703050 |
| ssa01 | 140765116 | LOC106565903 | 0.02706 | NA529856_NC_059442.1_140765116 |
| ssa01 | 140730915 | LOC106565903 | 0.02756 | NA529791_NC_059442.1_140730915 |
| ssa15 | 64723913 | LOC106571912 | 0.02756 | NA5948767_NC_059456.1_64723913 |
| ssa09 | 1288851 | LOC106610641 | 0.02785 | NA2958140_NC_059450.1_1288851 |
| ssa07 | 51965584 | LOC100380729 | 0.02837 | NA2795230_NC_059448.1_51965584 |
| ssa09 | 78632209 | si:zfos-2326c3.2 | 0.03046 | NA3230922_NC_059450.1_78632209 |
| ssa09 | 132652875 | cadm1b | 0.03078 | NA3414538_NC_059450.1_132652875 |
| ssa16 | 4115755 | LOC106573059 | 0.03286 | NA6140278_NC_059457.1_4115755 |
| ssa01 | 140767540 | LOC106565903 | 0.0336 | NA529857_NC_059442.1_140767540 |
| ssa01 | 140742351 | LOC106565903 | 0.03412 | NA529823_NC_059442.1_140742351 |
| ssa01 | 118613520 | ryr2a | 0.03419 | NA458621_NC_059442.1_118613520 |
| ssa01 | 118613522 | ryr2a | 0.03419 | NA458622_NC_059442.1_118613522 |
| ssa15 | 64731969 | LOC106571912 | 0.03419 | NA5948786_NC_059456.1_64731969 |
| ssa15 | 64717461 | LOC106571912 | 0.03644 | NA5948751_NC_059456.1_64717461 |
| ssa15 | 64717614 | LOC106571912 | 0.03673 | NA5948752_NC_059456.1_64717614 |
| ssa07 | 51965581 | LOC100380729 | 0.03684 | NA2795228_NC_059448.1_51965581 |
| ssa01 | 140832594 | LOC106565929 | 0.03841 | NA529996_NC_059442.1_140832594 |
| ssa11 | 45560553 | crybgx | 0.03845 | NA4180613_NC_059452.1_45560553 |
| ssa18 | 55657076 | LOC106577504 | 0.03845 | NA6927187_NC_059459.1_55657076 |
| ssa18 | 55657077 | LOC106577504 | 0.03845 | NA6927188_NC_059459.1_55657077 |
| ssa18 | 55657081 | LOC106577504 | 0.03845 | NA6927189_NC_059459.1_55657081 |
| ssa18 | 55657083 | LOC106577504 | 0.03845 | NA6927190_NC_059459.1_55657083 |
| ssa17 | 63643878 | ppfia2 | 0.03911 | NA6644296_NC_059458.1_63643878 |
| ssa15 | 64731206 | LOC106571912 | 0.03957 | NA5948781_NC_059456.1_64731206 |
| ssa28 | 36637557 | lrit1b | 0.04021 | NA9556155_NC_059469.1_36637557 |
| ssa10 | 55608902 | LOC106594307 | 0.04027 | NA3760124_NC_059451.1_55608902 |
| ssa25 | 22570991 | sema5ba | 0.04027 | NA8789068_NC_059466.1_22570991 |
| ssa17 | 63640581 | ppfia2 | 0.04152 | NA6644201_NC_059458.1_63640581 |
| ssa12 | 25770288 | LOC106588738 | 0.04383 | NA4555876_NC_059453.1_25770288 |
| ssa15 | 64721288 | LOC106571912 | 0.04397 | NA5948758_NC_059456.1_64721288 |
| ssa15 | 64735882 | LOC106571912 | 0.04428 | NA5948794_NC_059456.1_64735882 |
| ssa17 | 63641325 | ppfia2 | 0.04459 | NA6644203_NC_059458.1_63641325 |
| ssa01 | 140745756 | LOC106565903 | 0.04554 | NA529830_NC_059442.1_140745756 |
| ssa09 | 78642797 | si:zfos-2326c3.2 | 0.04554 | NA3230954_NC_059450.1_78642797 |
| ssa01 | 140770192 | LOC106565903 | 0.04743 | NA529864_NC_059442.1_140770192 |
| ssa09 | 1234107 | bahd1 | 0.04743 | NA2957842_NC_059450.1_1234107 |
| ssa15 | 64735973 | LOC106571912 | 0.04743 | NA5948795_NC_059456.1_64735973 |
| ssa17 | 63533446 | ppfia2 | 0.0475 | NA6643702_NC_059458.1_63533446 |
| ssa17 | 63780270 | lhfpl3 | 0.04857 | NA6644970_NC_059458.1_63780270 |
| ssa12 | 25770838 | LOC106588738 | 0.04876 | NA4555880_NC_059453.1_25770838 |
| ssa12 | 25771178 | LOC106588738 | 0.04876 | NA4555881_NC_059453.1_25771178 |
| ssa01 | 140834761 | LOC106565929 | 0.0498 | NA530002_NC_059442.1_140834761 |
| ssa02 | 3341706 | LOC106597444 | 0.04988 | NA682678_NC_059443.1_3341706 |
| ssa09 | 78725028 | LOC106611984 | 0.04988 | NA3231256_NC_059450.1_78725028 |
| ssa12 | 90579679 | LOC106566123 | 0.04988 | NA4768409_NC_059453.1_90579679 |
| ssa17 | 63529628 | ppfia2 | 0.04988 | NA6643683_NC_059458.1_63529628 |

**Table S3** Overlapping-gene associations with adult return timing in seven populations of North American Atlantic salmon (*Salmo salar*) using lc-WGS and corrected for population structure (K = 20), age-at-maturity and sex. Ordered according to significance according to the Benjamini-Hochberg false discovery rate correction (FDR_BH).

| **Chromosome** | **Position** | **gene** | **FDR_BH** | **SNP** |
| --- | --- | --- | --- | --- |
| ssa17 | 63719955 | golgb1 | 0.002658 | NA6644706_NC_059458.1_63719955 |
| ssa17 | 7699731 | LOC106575122 | 0.002931 | NA6457527_NC_059458.1_7699731 |
| ssa17 | 63638643 | ppfia2 | 0.003153 | NA6644198_NC_059458.1_63638643 |
| ssa17 | 63779053 | LOC106576354 | 0.01163 | NA6644958_NC_059458.1_63779053 |
| ssa07 | 14731264 | LOC106608688 | 0.01662 | NA2641300_NC_059448.1_14731264 |
| ssa17 | 7700259 | LOC106575122 | 0.01662 | NA6457530_NC_059458.1_7700259 |
| ssa17 | 7703301 | LOC106575122 | 0.01662 | NA6457549_NC_059458.1_7703301 |
| ssa17 | 63641852 | ppfia2 | 0.01662 | NA6644209_NC_059458.1_63641852 |
| ssa17 | 63703696 | LOC106564527 | 0.01662 | NA6644652_NC_059458.1_63703696 |
| ssa17 | 63719798 | golgb1 | 0.01662 | NA6644705_NC_059458.1_63719798 |
| ssa17 | 63777196 | LOC106576354 | 0.01662 | NA6644947_NC_059458.1_63777196 |
| ssa17 | 63573783 | ppfia2 | 0.01694 | NA6643819_NC_059458.1_63573783 |
| ssa17 | 63700162 | llph | 0.01699 | NA6644643_NC_059458.1_63700162 |
| ssa17 | 63704186 | LOC106564527 | 0.01699 | NA6644657_NC_059458.1_63704186 |
| ssa17 | 64737682 | LOC106576423 | 0.01699 | NA6649709_NC_059458.1_64737682 |
| ssa17 | 7700578 | LOC106575122 | 0.01843 | NA6457533_NC_059458.1_7700578 |
| ssa17 | 7700980 | LOC106575122 | 0.01843 | NA6457536_NC_059458.1_7700980 |
| ssa17 | 63778720 | LOC106576354 | 0.01843 | NA6644956_NC_059458.1_63778720 |
| ssa17 | 63637324 | ppfia2 | 0.01859 | NA6644191_NC_059458.1_63637324 |
| ssa17 | 63527371 | ppfia2 | 0.02206 | NA6643679_NC_059458.1_63527371 |
| ssa17 | 7702976 | LOC106575122 | 0.02364 | NA6457544_NC_059458.1_7702976 |
| ssa17 | 63641554 | ppfia2 | 0.02364 | NA6644208_NC_059458.1_63641554 |
| ssa17 | 7700792 | LOC106575122 | 0.02498 | NA6457535_NC_059458.1_7700792 |
| ssa17 | 63637610 | ppfia2 | 0.02521 | NA6644193_NC_059458.1_63637610 |
| ssa09 | 132681362 | LOC106613023 | 0.02529 | NA3414658_NC_059450.1_132681362 |
| ssa09 | 132681456 | LOC106613023 | 0.02529 | NA3414659_NC_059450.1_132681456 |
| ssa17 | 7686510 | LOC106575122 | 0.02529 | NA6457463_NC_059458.1_7686510 |
| ssa17 | 7687786 | LOC106575122 | 0.02529 | NA6457466_NC_059458.1_7687786 |
| ssa17 | 7688468 | LOC106575122 | 0.02529 | NA6457468_NC_059458.1_7688468 |
| ssa17 | 7703319 | LOC106575122 | 0.02529 | NA6457550_NC_059458.1_7703319 |
| ssa15 | 64737257 | LOC106571912 | 0.02689 | NA5948796_NC_059456.1_64737257 |
| ssa15 | 64737809 | LOC106571912 | 0.02689 | NA5948817_NC_059456.1_64737809 |
| ssa05 | 68213549 | si:dkey-262k9.2 | 0.02793 | NA2099883_NC_059446.1_68213549 |
| ssa15 | 64731197 | LOC106571912 | 0.02793 | NA5948780_NC_059456.1_64731197 |
| ssa17 | 63638201 | ppfia2 | 0.02793 | NA6644196_NC_059458.1_63638201 |
| ssa17 | 63557681 | ppfia2 | 0.0286 | NA6643790_NC_059458.1_63557681 |
| ssa17 | 63570778 | ppfia2 | 0.02938 | NA6643810_NC_059458.1_63570778 |
| ssa20 | 56476232 | trip12 | 0.02987 | NA7581496_NC_059461.1_56476232 |
| ssa17 | 63731042 | golgb1 | 0.031 | NA6644748_NC_059458.1_63731042 |
| ssa02 | 64384831 | LOC106590330 | 0.03522 | NA902420_NC_059443.1_64384831 |
| ssa15 | 64734747 | LOC106571912 | 0.03562 | NA5948791_NC_059456.1_64734747 |
| ssa09 | 1277607 | LOC106610641 | 0.03566 | NA2958089_NC_059450.1_1277607 |
| ssa17 | 63537932 | ppfia2 | 0.03581 | NA6643736_NC_059458.1_63537932 |
| ssa01 | 140768182 | LOC106565903 | 0.04012 | NA529861_NC_059442.1_140768182 |
| ssa09 | 78633597 | LOC106611981 | 0.04137 | NA3230928_NC_059450.1_78633597 |
| ssa15 | 64727986 | LOC106571912 | 0.04336 | NA5948775_NC_059456.1_64727986 |
| ssa15 | 64728472 | LOC106571912 | 0.04336 | NA5948779_NC_059456.1_64728472 |
| ssa11 | 40515150 | LOC106591088 | 0.0441 | NA4162858_NC_059452.1_40515150 |
| ssa16 | 74348012 | LOC123727767 | 0.0441 | NA6376300_NC_059457.1_74348012 |
| ssa17 | 63778939 | LOC106576354 | 0.04437 | NA6644957_NC_059458.1_63778939 |
| ssa09 | 132685519 | LOC106613023 | 0.04513 | NA3414671_NC_059450.1_132685519 |
| ssa01 | 140730915 | LOC106565903 | 0.04543 | NA529791_NC_059442.1_140730915 |
| ssa01 | 140725011 | LOC106565903 | 0.04848 | NA529780_NC_059442.1_140725011 |
| ssa01 | 140765116 | LOC106565903 | 0.04848 | NA529856_NC_059442.1_140765116 |
| ssa01 | 140767540 | LOC106565903 | 0.04848 | NA529857_NC_059442.1_140767540 |
| ssa09 | 152196151 | kcnab1a | 0.04848 | NA3498079_NC_059450.1_152196151 |
| ssa10 | 55608902 | LOC106594307 | 0.04848 | NA3760124_NC_059451.1_55608902 |
| ssa11 | 45560553 | LOC106562861 | 0.04848 | NA4180613_NC_059452.1_45560553 |
| ssa17 | 7689065 | LOC106575122 | 0.04848 | NA6457495_NC_059458.1_7689065 |
| ssa17 | 63534303 | ppfia2 | 0.04848 | NA6643706_NC_059458.1_63534303 |
| ssa17 | 63535438 | ppfia2 | 0.04848 | NA6643708_NC_059458.1_63535438 |
| ssa17 | 64221053 | LOC106576364 | 0.04848 | NA6647485_NC_059458.1_64221053 |
| ssa17 | 64221054 | LOC106576364 | 0.04848 | NA6647486_NC_059458.1_64221054 |
| ssa24 | 8838209 | araf | 0.04848 | NA8528726_NC_059465.1_8838209 |
| ssa25 | 22570987 | LOC106586418 | 0.04848 | NA8789067_NC_059466.1_22570987 |
| ssa01 | 118613520 | ryr2a | 0.04998 | NA458621_NC_059442.1_118613520 |
| ssa01 | 118613522 | ryr2a | 0.04998 | NA458622_NC_059442.1_118613522 |

**Table S4** Results from the enrichment of genes associated with adult return timing in seven populations of North American Atlantic salmon. Using the Ssal_v3.1 Atlantic salmon genome as the genomic background, ShinyGO 0.80 (Ge et al., 2020) identified over-represented genetic pathways by searching two pathway databases: KEGG and GO Biological Processes.

| **Pathway database** | **Enrichment FDR** | **nGenes** | **Pathway Genes** | **Fold Enrichment** | **Pathway** | **Genes** |
| --- | --- | --- | --- | --- | --- | --- |
| GO Biological Processes | 0.039 | 1 | 17 | 276.7235294 | GO:0008016 reg. of heart contraction | chrm2a |
| GO Biological Processes | 0.039 | 1 | 23 | 204.5347826 | GO:0003015 heart proc. | chrm2a |
| GO Biological Processes | 0.039 | 1 | 23 | 204.5347826 | GO:0060047 heart contraction | chrm2a |
| GO Biological Processes | 0.048 | 1 | 38 | 123.7973684 | GO:1903522 reg. of blood circulation | chrm2a |
